# Harnessing DNA Replication Stress for Novel Cancer Therapy

**DOI:** 10.3390/genes11090990

**Published:** 2020-08-25

**Authors:** Huanbo Zhu, Umang Swami, Ranjan Preet, Jun Zhang

**Affiliations:** 1 Division of Medical Oncology, Department of Internal Medicine, University of Kansas Medical Center, 3901 Rainbow Blvd, Kansas City, KS 66160, USA; hzhu2@kumc.edu (H.Z.); rpreet@kumc.edu (R.P.); 2 Division of Oncology, Department of Internal Medicine, Huntsman Cancer Institute, University of Utah, 2000 Circle of Hope, Salt Lake City, UT 84112, USA; umang.swami@hci.utah.edu; 3 Department of Cancer Biology, University of Kansas Cancer Center, 3901 Rainbow Blvd, Kansas City, KS 66160, USA

**Keywords:** DNA replication stress, ATR, Chk1, PARP, WEE1, MELK, TLK, NAE, NEDD8, DNA damage response

## Abstract

DNA replication is the fundamental process for accurate duplication and transfer of genetic information. Its fidelity is under constant stress from endogenous and exogenous factors which can cause perturbations that lead to DNA damage and defective replication. This can compromise genomic stability and integrity. Genomic instability is considered as one of the hallmarks of cancer. In normal cells, various checkpoints could either activate DNA repair or induce cell death/senescence. Cancer cells on the other hand potentiate DNA replicative stress, due to defective DNA damage repair mechanism and unchecked growth signaling. Though replicative stress can lead to mutagenesis and tumorigenesis, it can be harnessed paradoxically for cancer treatment. Herein, we review the mechanism and rationale to exploit replication stress for cancer therapy. We discuss both established and new approaches targeting DNA replication stress including chemotherapy, radiation, and small molecule inhibitors targeting pathways including ATR, Chk1, PARP, WEE1, MELK, NAE, TLK etc. Finally, we review combination treatments, biomarkers, and we suggest potential novel methods to target DNA replication stress to treat cancer.

## 1. Introduction

Precise, accurate, and error-free DNA duplication of the entire cell genome through DNA replication is necessary for the continuation of controlled, cellular proliferation [1]. It is a tightly regulated process comprising a vast number of signaling pathways that ensure that the genome is replicated only once with high fidelity [1]. To maintain genomic integrity, cells deploy the DNA damage response (DDR) system, a collective term for various signaling molecules and enzymes produced by more than 450 genes [2] which either activate DNA repair or induce cellular senescence or apoptosis [3].

Because of mutations including oncogenic activation or tumor suppressor gene inactivation, the DDR system may fail to safeguard genomic integrity and fidelity, and DNA replication may persist despite uncorrected DNA errors in response to proliferation signals leading to replication stress [3]. DNA replication stress is described as an alteration in error-free DNA replication including a slowdown of DNA synthesis and stalling of replication forks leading to genomic instability [3]. However, further enhancing replication stress in cancer cells can paradoxically lead to cell death through the induction of “mitotic catastrophe”—a type of cell death caused by premature or inappropriate entry of cells into mitosis prior to the completion of DNA synthesis—therefore offering a novel approach to treat cancer.

In this review, we explore the current strategies and summarize our current understanding of ways to harness replication stress to treat cancer. We focus on emerging approaches and propose novel ideas and biomarkers to predict responses.

## 2. Underlying Mechanisms of Replication Stress and Rationale in Cancer Therapy

Human DNA has daily exposure to a variety of insults which are both exogenous and endogenous in nature. Exogenous insults include ultraviolet radiation, ionizing radiation, genotoxic chemicals, and environmental stresses. While endogenous stresses include reactive oxygen species (ROS), spontaneous disintegration of chemical bonds, replication errors, DNA base mismatches, topoisomerase-DNA complexes, DNA methylation etc. [4]. In normal cells, damaged DNA is repaired by various mechanisms including base-excision repair (BER), nucleotide-excision repair (NER), mismatch repair (MMR), homologous recombination (HR), and non-homologous end-joining (NHEJ). In general, single-strand breaks (SSBs) are repaired by BER, NER, and MMR while double-strand breaks (DSBs) are repaired by HR and NHEJ [5]. If DNA repair is not possible, cell cycle progression through G2-M phase is blocked, and the cells enter senescence or apoptosis [3,6].

However, a faulty DDR system or altered checkpoints along with persistent growth signaling can lead to the replication of damaged DNA, thus, leading to DNA synthesis slowdown and/or replication fork stalling, characteristic of DNA replication stress [3,6,7]. “Fork stalling” can occur because of multiple reasons including, limited substrate availability (e.g., shortage of histones or deoxyribonucleotide triphosphates (dNTPs), abnormal DNA secondary structures or topology, RNA-DNA hybrids (R-loops), DNA-protein crosslinks, torsional stress, and cessation of DNA polymerase activity etc., [8,9]. Stalled forks initiate the firing of neighboring dormant origins during active replicative mini-chromosome maintenance (MCM) so that they can continue unwinding DNA for a few hundred base pairs downstream, which exposes single-stranded DNA (ssDNA) [3,8]. ssDNAs recruit/activate Ataxia telangiectasia and Rad3-related protein (ATR) through ATR interacting protein (ATRIP) which leads to phosphorylation of multiple targets including checkpoint kinase 1 (Chk1) [3,8]. All these events help to reduce replication stress [3].

In normal cells, ATR in combination with other DDR proteins and checkpoint molecules, prevent untimely mitosis of cells harboring damaged DNA [10]. But in cancer cells with replicative stress, stalled forks exist and multiple origins are fired, leading to dNTP pool depletion [11]. This further enhances replication stress leading to the generation of more non-progressive forks [11]. When there is an excess of ssDNA, it depletes available replication protein A (RPA), which causes forks to collapse leading to double-strand DNA (dsDNA) break generation [3,9,11,12]. If these cells enter mitosis their damaged, non-replicated DNA can cause “mitotic catastrophe” leading to cell death [13,14]. However, it needs to be noted that even though replicative stress is present in cancer cells it occurs only at low to mild levels as its excess can cause mitotic catastrophe [6,7,15].

**Figure 1:**
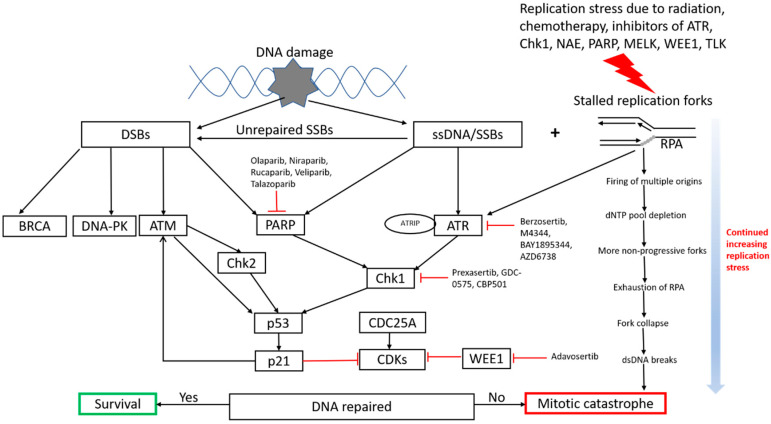
Current and potential strategies to target DNA replication stress. The major pathways involved in DNA damage response and replication stress are illustrated in this flowchart underlying the details of replication stress and cancer therapy targets. (ATR: Ataxia telangiectasia and Rad3-related protein; ATRIP: Ataxia telangiectasia and Rad3-related protein interacting protein; RPA: replication protein A; ATM: ataxia telangiectasia mutated; DNA-PK: DNA-dependent protein kinase; CDK: cyclin-dependent kinase; DSBs: double strand breaks; SSBs: single strand breaks; ssDNA: single-stranded DNA; dNTP: deoxyribonucleotide triphosphates).

The persistence of replicative stress is observed almost exclusively in precancerous and cancer cells and is rarely seen in normal cells even with rapid proliferation [7], which offers potential therapeutic selectivity in cancer cells. This is likely due to multiple reasons including oncogene activation, inactivation of tumor suppressor genes, higher levels of ROS, defective DDR system etc., in cancer cells [3]. However, as discussed even though replicative stress causes genomic instability and mutations which are considered a hallmark of cancer [16], it can be paradoxically harnessed to treat cancer by exploiting it to push cancer cells into replicative catastrophe [3]. This can theoretically be done by either targeting DNA synthesis, replication, repair, promoting cells to enter S phase or pushing their premature entry into M phase [3]. Figure 1 summarizes the major pathways involved in DNA replication stress and current and potential strategies to target them.

## 3. Potential Pathways to Modulate Replication Stress for Cancer Treatment

### 3.1. Traditional Approaches—Radiation and Chemotherapy

Radiation-induced DNA damage ranges from base and sugar damage to cross-links, SSBs and DSBs [17] which interfere with DNA replication. However, DSBs are mostly responsible for radiation lethality to cells [17]. 

Multiple chemotherapeutic agents induce and modulate DNA replication stress in their own unique ways. Some of them like DNA cross-linking agents, topoisomerase inhibitors and alkylating agents act primarily by causing DNA damage. This further induces and enhances replication stress in cancer cells, while agents targeting nucleotide synthesis and inhibiting DNA polymerases act by thwarting the activity of replisomes thereby, increasing replication stress and activating the replication stress response [8]. As discussed in Table 1, some alkylating agents (e.g., cyclophosphamide, ifosfamide etc.,) and alkylating-like agents (platinum compounds such as cisplatin, carboplatin etc.,) cause inter- and intra-strand cross-links between DNA bases [18,19]. Inter-strand cross-links hinder DNA unwinding [20] and intra-strand cross-links misincorporate nucleotides and produce alterations in template strand [21]. These cross-links can delay or stall the progression of replication forks and lead to and increased replication stress [3,8].

Chemotherapies like gemcitabine, cytarabine, fludarabine, 5-fluorouracil, and hydroxyurea slow or stall replication fork progression by directly reducing dNTP pools leading to direct inhibition of DNA synthesis and/or by inhibiting DNA polymerases. Nucleoside analogs like gemcitabine, fludarabine, and cytarabine are incorporated into replicated DNA (or RNA) strand after converting into triphosphates and inhibit replication or transcription elongation [22]. Gemcitabine (irreversibly) and hydroxyurea (reversibly) inhibit ribonucleotide reductase [23], while 5-fluorouracil inhibits thymidylate synthetase [24]. This also depletes the available dNTP pool, the building block of DNA strands which slows and stalls replication fork progression [3].

Topoisomerases control DNA topology by catalyzing the cleavage and resealing of one or both DNA strands thereby, managing supercoils and replication [25]. Topoisomerase inhibitors can induce replication stress, they can either physically hinder replication forks [26,27] or induce fork reversal as with topoisomerase I inhibitors (irinotecan and topotecan) [28] or activate Chk1 as with topoisomerase II inhibitors (etoposide and doxorubicin) [29]. 

### 3.2. New Emerging Approaches and Strategies

#### 3.2.1. ATR-Chk1 Pathway

ATR along with ataxia telangiectasia mutated (ATM) and the DNA-dependent protein kinase (DNA-PK), are the key components of the DDR system [30]. ATM and DNA-PK primarily respond to DSBs while ATR responds to replication stress including ssDNA generated at stalled replication forks or resected DSBs [7,30,31]. Table 2 summarizes the current clinical trials of novel agents targeting DNA replication stress. 

Cancer cells can tolerate relatively elevated levels of replication stress because of a proficient response system that includes the ATR-Chk1 pathway [32]. The activation of ATK-Chk1 is crucial in suppressing further replicative stress and therefore helps cancer cells avoid deleterious events such as mitotic catastrophe. Inhibiting ATR or Chk1 can sensitize cancer cells to therapies harnessing replication stress and therefore combining ATR/Chk1 inhibitors to therapies inducing replication stress seems to be a logical approach [32].

Inhibition of the ATR/Chk1 pathway can induce synthetic lethality in cells with high levels of oncogene-induced replication stress such as cells with oncogenic Ras, APOBEC3A, or c-MYC overexpression as well as in cells deficient with p53, ATM, or POLD1 [33,34,35,36,37,38]. Synthetic lethality with ATR inhibitors has also been observed with AT-rich interaction domain 1A (ARID1A) mutant tumors, mutations causing accumulation of R loops (RNA-DNA hybrids) like U2AF1 (S34F) and with cancers overexpressing oncogenic SS18-SSX fusion proteins like synovial sarcoma [36,39,40,41]. 

Schisandrin B, a natural product, was the first ATR specific inhibitor, but the clinical application was restricted because of the requirement of a very high dose [42]. Though multiple agents are in preclinical development only a few agents namely VX-803 (M-4344), VX970 (berzosertib, M6620), AZD6738, BAY1895344 have reached clinical trials [43].

Chk1 also suppresses CDK activity through negative regulation of CDC25A phosphatase to manage precise activation of replication origins during S phase [44,45]. As compared to ATR inhibition, Chk1 inhibition is more detrimental to cycling cells [46]. 

Development of Chk1 inhibitors have advanced further for multiple reasons including a wider, non-specific effect of ATR inhibition than Chk1 inhibition, difficulties with purification of active ATR protein and enzymatic analysis due to its large size and the lack of a crystallographic or cyro-EM structure and hence unawareness about possible allosteric binding sites for drug design and lack of standardized high throughput assays for analysis of ATR inhibitors [47,48,49,50].

UCN-01 (7-hydroxystaurosporine) was the first Chk1 inhibitor. The development of which hit roadblocks because of the lack of specificity and unusual pharmacokinetic features including low distribution volume, systemic clearance, and long elimination half-life (>200 h), likely due to high affinity to α-1 acid glycoprotein [51,52]. Thereafter, multiple other agents including XL844, CBP501, AZD7762, LY2603618, MK-8776 (SCH 900776), PF-00477736, LY2606368 (Prexasertib) reached clinical trials but are yet to show valuable activity and efficacy [52].

#### 3.2.2. PARP Inhibitors

Poly ADP-ribose polymerase (PARP) family comprises 18 enzymes, amongst them, PARP1 is the most well studied [3,53]. PARP1 is a major component of DNA damage repair pathway and its role in DNA repair has been covered in detail in an excellent review by Ray Chaudhuri and André Nussenzweig [53]. In brief, PARP1 activates SSB repair, is involved in ssDNA nick repairs, promotes nucleotide excision repair, acts as a sensor of dsDNA breaks, and modulates chromatin structure in response to DNA damage [53]. It is also involved in the HR and NHEJ pathways [53]. It recruits MRE11 to initiate the end processing required for replication restart and is also implicated in the recruitment of other HR proteins [53]. PARP1 has been shown to be involved in the recruitment of DNA repair and checkpoint proteins at sites of DNA damage, enhancing Chk1 activation and its binding at stalled replication forks, which is required for replication restart [54,55,56]. It also regulates the rate of DNA replication fork progression during DNA replication stress and its inhibition during replication stress leads to abrogation of fork slowdown [53]. Therefore, inhibition of PARP1 increases replication stress [3].

However, even though high PARP-1 expression is observed in many tumors [57], its inhibition alone has limited efficacy [58]. But it becomes lethal to tumors with a defect in DNA repair pathway as seen in breast cancer gene (BRCA)-deficient tumors [58]. BRCA1 and BRCA2 proteins are essential for the repair of dsDNA breaks via HR repair, and cells with deficient or altered HR repair pathway depend on non-conservative forms of DNA repair like inaccurate NHEJ [58]. 

Deficiency of genes involved in HR repair pathways like RAD51, RAD54, DSS1, RPA1, NBS1, ATR, ATM, Chk1, Chk2, FANCD2, FANCA, or FANCC also induce sensitivity to PARP inhibitors [59]. This provides a potentially unique opportunity for PARP therapies in cancers with loss-of-function mutations in these genes. 

Sporadic tumors might also develop a phenocopy of BRCA1 or BRCA2 mutation where HR repair defects exist in tumor because of other reasons like BRCA1 promoter hypermethylation, amplification of EMSY etc., without germline BRCA1 or BRCA2 mutation, a phenomenon known as BRCAness [60]. PARP inhibitors might also provide a rational approach to target the tumors with BRCAness [60]. 

This synthetic lethality with PARP1 inhibitors in BRCA or HR-deficient tumors is thought to be due to various reasons. First, if ssDNA breaks are persistent, they will convert to DSB and eventually need DSB repair (HR and NHEJ) pathways. Therefore, BRCA or HR-deficient cancer cells need NHEJ for DSB repair which is PARP1 dependent and its inhibition can drive them toward apoptosis [61,62]. Second, both BRCA2 and PARP1 prevent Mre11-dependent degradation of stalled replication forks [63]. Actually, BRCA1, BRCA2, PARP1, and PARP2 all prevent or mediate stalled folk degradation [64,65]. Third, PARP inhibitors trap PARP1 and PARP2 enzymes at damaged DNA site which obstructs other DNA repair proteins [66]. Fourth, inhibiting PARP during replicative stress makes cells depend on HR for DNA repair and leads to synthetic lethality in BRCA or HR-deficient tumors [3,61]. 

Nicotinamide was the first known naturally occurring PARP inhibitor and 3-aminobenzamide was the first synthetic PARP inhibitor [67,68]. Iniparib (BSI-201) was the first PARP inhibitor to reach phase III trial but failed to meet its primary endpoint [69]. Thereafter multiple PARP inhibitors including olaparib, rucaparib, niraparib, veliparib, and talazoparib have reached clinical trials and four of this niraparib, rucaparib, Olaparib, and talazoparib have received FDA approval (Table 1) [58]. 

PARPs (in particular PARP1), along with RECQ-like (RECQL) helicases, including RECQL1, WRN, BLM, RECQL4, and RECQL5, represent two central quality control systems to preserve genome integrity in mammalian cells. Both are involved in the control of DNA repair, telomere maintenance, and replicative stress [70]. RECQL1, which is first discovered, provides several potential functional overlaps with PARP1 and other RECQL helicases due to its involvement in replication, DNA repair, and telomere maintenance [71]. WRN, which is also called RECQL2, is revealed to have functional and biochemical relevant interactions with PARP1 on a biochemical, cellular, and organismal level [70]. BLM, also known as RECQL3, plays important roles in HR where it is involved in DNA end-resection as well as in the subsequent branch migration and resolution of Holliday junctions (HJ) or double HJ (dHJ), which may contribute to HR fidelity and suppresses crossover reactions [72,73]. As all five RECQL helicases physically or functionally interact with PARP1 and/or its enzymatic product poly (ADP-ribose) (PAR), the cooperative function of RECQLs and PARP1 is crucial to maintaining genome integrity. In fact, RECQ1 is found to be required for cellular resistance to replication stress [74], and loss-of-function mutations in the RECQ1 gene are associated with increased susceptibility to breast cancer [75], as well as enhanced sensitivity to DNA damaging agents [76].

Other PARP members, such as PARP10, were recently found to be overexpressed in various tumors and promote cellular proliferation [77]. Its overexpression alleviates replication stress and is suggested to promote the restart of replication forks [77]. PARP10 can be another novel target to further enhance DNA replicative stress for cancer therapies.

#### 3.2.3. Other Targets That Are Relevant to DNA Replication Stress

Overexpression and upregulation of HR proteins like RAD51 are commonly encountered in malignant cells [78]. RAD51 is involved in HR-mediated repair of DSBs and promotion of replication fork stability during replication stress and therefore can contribute to tumor resistance to DNA-damaging therapies [78]. Therefore, targeting proteins involved in HR repair like RAD51 seems promising for future investigations [78,79]. RAD51 inactivation appeared to increase ATR/Chk1-mediated replication signaling and inhibition of ATR or Chk1 in this scenario can provide another opportunity for exploring synthetic lethality [80].

Many cancer cells appear to depend on constitutive hyper-signaling of ATR to upregulate HR proteins to overcome replication stress. Chronic long-term inhibition of ATR signaling can severely impair homologous recombination (HR)-mediated DNA repair and abundance of HR proteins in cancer cells [81]. This can mimic “BRCAness” and can sensitize HR-proficient cancer cells to PARP inhibitors after chronic treatment which can provide a basis for the synergistic combination of ATR and PARP inhibitors in this setting [81].

Cyclin-dependent kinase 12 (CDK12) is a member of cyclin-dependent kinase (CDK) family of serine/threonine protein kinases which can regulate transcriptional and post-transcriptional processes, and therefore modulate multiple cellular functions [82]. CDK12 upregulates genes involved in the response to DNA damage and stress. Mutations in CDK12 gene have been observed in multiple cancer types [82]. CDK12 inhibition is predicted to not only inhibit tumor growth but can lead to synthetic lethality with MYC, EWS/FL1, and PARP/Chk1 inhibition [82].

Tousled-like kinases 1 and 2 (TLK1/TLK2) are nuclear serine/threonine kinases which are regulated by Chk1 [83]. TLK1/2 are required for DNA replication and replication-coupled nucleosome assembly [84]. Their inhibition can lead to the stalling of the replication forks and ssDNA accumulation, ultimately leading to increased replication stress [84]. In fact, depletion of TLKs was found synthetic lethal with checkpoint inactivation and PARP inhibitors [84].

WEE1 is a nuclear serine/threonine kinase that inhibits CDK1 and 2 which activates G2/M cell cycle checkpoint leading to the inhibition of cell entry into mitosis [85]. WEE1 inhibition leads to enhancement of CDK activity causing unscheduled entry into M phase even if DNA replication is incomplete or defective, causing unchecked firing of replication origins, nucleotide shortage translating into increased replication stress, and death from mitotic catastrophe [3,85,86]. Currently, adavosertib (AZD1775, MK-1775) is the only WEE1 inhibitor undergoing clinical development [86]. Maternal embryonic leucine zipper kinase (MELK), a serine/threonine protein kinase that belongs to the sub-family of AMP-activated serine/threonine protein kinases, has appeared as a target for decreasing replication stress threshold [87]. MELK plays a major role in various cellular and biological processes including DNA repair [88]. OTS167 is a MELK inhibitor which is currently undergoing clinical development (NCT02926690, NCT01910545, NCT02795520). However, the role of MELK as a cancer target is currently controversial [89].

Neddylation, a ubiquitin-like modification can reduce replication stress [90]. NEDD8 is one of the most studied ubiquitin-like protein which plays a critical role in mediating the ubiquitination of numerous cullin-RING ligases substrate proteins involved in cell cycle progression and survival [91]. Inhibition of neddylation leads to the stabilization of the substrates of these ubiquitin ligases including DNA replication factor CDT1, which forms a complex with the replication-licensing factor geminin, causing DNA to replicate more than once per S phase, therefore rapidly consuming dNTP [92,93]. Depleting CDT1 inhibitor geminin can increase origin firing to induce re-replication in cancer cells leading to DNA damage and apoptosis [7]. Pevonedistat (TAK-924/MLN4924) is a novel inhibitor of NEDD8-activating enzyme currently undergoing multiple phase I/II trials (Table 2). It causes CDT1 stabilization, re-replication, senescence, and apoptosis in cancer cells [93]. TAS4464, another highly potent NEDD8-activating enzyme inhibitor, underwent a phase I/II study in multiple myeloma and lymphoma but the study has been terminated due to business reasons (NCT02978235).

Apart from ATR/Chk1 pathway, ATM/Chk2 and DNA-PK pathways are also activated during DNA replication stress likely because of secondary DSBs and obstructing these pathways with inhibitors of ATM, Chk2, DNA-PK, and their downstream proteins can potentiate replication stress [94,95].

## 4. Potential Combination Treatment Approaches

Replication stress pathway includes a concoction of multiple checkpoints and proteins that are exploited in unique ways by various agents discussed above to target cancer cells. Therefore, a combination approach of these agents appears to be an attractive option. For the last many years combination of chemotherapy with radiation has been considered the standard of care in neoadjuvant and adjuvant settings as well as part of definitive treatment for many cancers [96,97,98]. Similarly, combination chemotherapies, as well as chemoimmunotherapy combinations, have received approval for various cancers [99,100]. Combining immunotherapies with agents targeting DNA repair like ATM, ATR, or DNA-PK inhibitors or PARP inhibitors present attractive options for combination therapies in selected cancers [101]. In fact, many clinical trials are currently in progress with these combinations [101].

The ATR-Chk1 pathway is activated by ssDNA and plays an important role in controlling replicative stress [3]. ATR inhibitors show enhanced activity with molecules capable of inducing replication stress [30] like inter-strand cross-linking agents (mitomycin C, cisplatin, carboplatin), nucleoside analogs (gemcitabine, cytarabine), PARP inhibitors and topoisomerase inhibitors (irinotecan, etoposide) in various cancer cell lines [30]. ATR inhibitors have a radiosensitizing effect which is more modest when compared to chemosensitizing agents [30,52]. ATR and PARP inhibition induces specific cytotoxicity in GBM cancer stem-like cells and negates radiation resistance [102]. ATR inhibitors have shown synergy with agents forcing premature mitotic entry like WEE1 inhibitors in mouse embryonic stem cells [103] and Chk1 inhibitors by further suppression of replication stress [30]. ATR inhibitors have also been shown to synergize with insulin-like growth factor 1 receptor tyrosine kinase inhibitor in breast cancer cell lines [104] and bromodomain and extra-terminal (BET) family inhibitors in melanoma and MYC-induced lymphoma [30,105,106].

PARP inhibitors have also shown synergy with radiation, various chemotherapies like alkylating agents, topoisomerase inhibitors, platinum compounds, EGFR-directed therapies, anti-vascular endothelial growth factor therapies, and PI3K inhibitors [3,57,58]. Similarly, WEE1 inhibitors have shown synthetic lethality with Chk1 inhibitors and in H3K36me3-deficient cancers [107,108]. WEE1 inhibitors are also predicted to be synthetically lethal in cells with defects in Fanconi Anemia and HR pathways [109]. Preclinical studies have shown synergy between WEE1 and PARP inhibitors in small-cell lung cancer [110].

Cisplatin has shown synergy with Chk1 and WEE1 inhibitors in preclinical models [85,111]. Gemcitabine has shown synergy with ATR, Chk1, WEE1, and NEDD8-activating enzyme inhibitors in preclinical models [112,113,114,115] but unexpected cardiotoxicity with Chk1 inhibitor AZD7762 in clinical trials [116,117]. Therefore, even though we have multiple combinations which have shown synergism or synthetic lethality in preclinical models, these novel combinations need careful evaluation in clinical setting due to unexpected toxicities.

In the era of cancer immunotherapy, it is very important to understand whether we can take advantage of DNA replicative stress to further enhance immunotherapy. It is known that DSBs can upregulate PD-L1 expression in cancer cells [118]. This upregulation requires ATM/ATR/Chk1 kinases and is augmented after DSBs when a specific DSB repair protein, BRCA2 or Ku70/80 is depleted [118]. Pembrolizumab, an anti PD-1 immunotherapy has been approved in unresectable or metastatic, microsatellite instability-high or mismatch repair deficient tumors [119]. In fact, defects in DNA repair such as DDR deficiency lead to genomic instability, higher tumor immunogenicity, greater mutational and neoantigen burden which leads to an improved response to immunotherapy [101]. Therefore, immunotherapy may prove to be beneficial in combination with agents modulating replication stress to treat cancer. However, caution needs to be taken as an intact DDR plays an important role in immunity [120], and therefore the optimal combination with various immunotherapy needs to be further determined [121].

## 5. Future Directions, Identification of Biomarkers and Resistance Mechanisms

Even though we have come a long way in understanding replication stress and a great deal of effort is being undertaken in harnessing it for cancer treatment, we have met with limited success. Therefore, there is a need to look beyond the conventional approaches. Further investigations need to be done to potentiate replication stress by combining it with other traditional pathways. It is well-known that cancer cells are in oxidative stress [122] and increasing reactive oxygen species levels can further enhance replicative stress due to the incorporation of oxidized nucleotides [122,123]. This approach can selectively target cancer cells, due to them being in a perennial prooxidative state as compared to normal cells [122]. It is also hypothesized that increasing reactive oxygen species in cancer stem cells may make them more radiosensitive [124]. Targeting chromatin with agents like histone deacetylase inhibitors increases replicative stress [125,126,127]. Moreover, synergism has been observed between histone deacetylase and WEE1 inhibitors [128]. This provides another rational combination to target replication stress. Other potential approaches include modulating cell death pathways like apoptosis, senescence, and autophagy as they all induce profound replication stress [3,129,130,131]. Theoretically, enhancing G1-S transition can also potentiate DNA replication stress as replication stress is only possible during DNA synthesis (S phase) but on the downside can lead to enhanced tumor growth too [3,7,132]. Incorporating damaged dNTPs in cancer cells has been suggested as a strategy to increase replication stress by targeting NUDT1 (MTH1), which prevents misincorporation of oxidized dNTPs during replication [7,133,134]. Other alternatives include depleting licensing factors like ORC1 to sensitize tumor cells to hydroxyurea and H_2_O_2_ [7,135]. Similar result was also observed following CDC6 depletion in KRAS positive cancer cells [136].

For successful targeting of replication stress, there is an urgent need for a set of biomarkers which cannot only provide an accurate and precise quantification of replication stress in cancer cells but can also be used for modulating treatment and guiding diagnosis, treatment, and prognosis. Advancements have been made in identifying patients for precision medicine treatment like BRCA mutations for PARP inhibitors [69]. With regards to measuring replication stress, possible biomarkers include measurement of proteins bound to stalled replication fork by immunofluorescence analysis (γH2AX, FANCD2, RPA etc.), DNA fiber assays for stalled replication fork, karyotype analysis, lagging DNA, ultra-fine anaphase DNA bridge analysis, micronuclei analysis, whole genome sequencing, SNP array analysis, microarray analysis, immunohistochemical analysis of proteins involved in replication stress or chromosome instability, chromatin immunoprecipitation for DNA replication and repair factors, bromodeoxyuridine staining for detection of ssDNA accumulation etc., [137,138,139]. Some of these novel methods can be utilized only in preclinical settings to evaluate the effectiveness of potential strategies in modulating replication stress, while others can be used on patient samples after validation [137]. However, the major need is for biomarkers that can monitor these effects in a non-invasive, cost-effective manner from peripheral blood in real-time. Current methods like immunohistochemical analysis of γH2AX do not differentiate replicative stress from general DNA damage, and apoptosis as measured by cleaved caspase or TUNEL which may reflect only the downstream consequences of replicative stress [3].

Another field of investigation is understanding the mechanisms of resistance to these novel agents. A prime example includes PARP inhibitors where multiple resistance mechanisms have been identified. These include restoration of BRCA1/2 function via secondary mutations [140], restoration of HR through somatic loss of NHEJ factor 53BP1 [141], decreased PARP expression levels [58], increased RAD51 activity [142], restoration of fork protection [143], and upregulation of ATP-binding cassette (ABC) transporters such as p-glycoprotein efflux pump and resultant higher rate of drug efflux [144]. To overcome these resistance mechanisms, the next generation of PARP inhibitors like AZD2461, which are poor substrates for drug transporters, are in development [145]. Other potential mechanisms to overcome resistance include employing inhibitors of multidrug resistance pumps like verapamil [146] and tariquidar [144]. Similar investigations are ongoing with ATR, Chk1, and Wee1 inhibitors [103,147].

## 6. Conclusions

In this review, we discuss current approaches to utilize DNA replication stress, along with underlying mechanisms and future directions. As we understand more about DDR pathways and cancer genomics, replication stress, and its modulation in cancer will be a major field of investigation and may help pave the way toward the personalized medicine in clinical practice. Multiple novel therapies targeting replication stress are in development and these novel molecules and biomarkers have the potential to advance the field with exciting impact on cancer treatment. The need will be to put them in the correct scenarios and understand the resistance mechanisms.

## Figures and Tables

**Table 1:** Agents approved by United States Food and Drug Administration targeting DNA replication stress.

Mechanism	Agents	Approved Indications
DNA mis incorporation/modification	Cyclophosphamide	HL, NHL, multiple myeloma, ALL, AML, breast cancer, CLL, CML, CLL, CML, mycosis fungoides, neuroblastoma, ovarian cancer, retinoblastoma
	Temozolomide	Anaplastic astrocytoma, glioblastoma multiforme
	Cisplatin	Bladder, testicular, ovarian cancer
	Carboplatin	Ovarian cancer
	Oxaliplatin	Colorectal cancer
Ribonucleotide reductase inhibitor	Gemcitabine	NSCLC, breast, ovarian, pancreatic cancer
	Clofarabine	ALL
	Fludarabine	CLL
	Cytarabine	ALL, AML, CML, meningeal leukemia, lymphomatous meningitis
	Hydroxyurea	CML, HNSCC
Thymidylate synthetase inhibition	5-Fluorouracil	Breast, colorectal, gastric, pancreatic cancer
	Capecitabine	Breast, colorectal caner
	Pemetrexed	NSCLC, malignant pleural mesothelioma
Topoisomerase I inhibitor	Irinotecan	Colorectal cancer, pancreatic cancer
	Topotecan	Cervical, ovarian, SCLC
Topoisomerase II inhibitor	Etoposide	SCLC, testicular cancer
	Doxorubicin	ALL, AML, HL, NHL, neuroblastoma, SCLC, soft tissue and bone sarcomas, Wilms tumor, thyroid, ovarian, breast, gastric, transitional cell bladder cancer
DNA methyltransferase inhibitor	Decitabine	MDS
Folic acid reductase inhibitor	Methotrexate	ALL, gestational trophoblastic disease, mycosis fungoides, NHL, osteosarcoma, head and neck, breast, lung cancer
DNA replication inhibitor	Nelarabine	T-cell ALL, T-cell lymphoblastic lymphoma
Blockage of synthesis and metabolism of purine nucleotides	Thioguanine	AML
Inhibition of nucleotide synthesis and nucleotide analogue incorporation	Trifluridine and Tipiracil Hydrochloride	Colorectal cancer
PARP inhibitors	Olaparib	BRCA-mutated, HER2-negative metastatic breast cancer, BRCA mutated advanced ovarian cancer, as maintenance treatment for recurrent epithelial OPFTC in patients experiencing a complete or partial response to platinum-based chemotherapy
	Rucaparib	BRCA mutated epithelial OPFTC after two or more chemotherapies, maintenance treatment of recurrent epithelial OPFTC that are in a complete or partial response to platinum-based chemotherapy
	Niraparib	Maintenance treatment of recurrent epithelial OPFTC in patients undergoing complete or partial response to platinum-based chemotherapy
	Talazoparib	Treatment of adult patients with deleterious or suspected deleterious germline breast cancer susceptibility gene (BRCA)-mutated (gBRCAm) human epidermal growth factor receptor 2 (HER2)-negative locally advanced or metastatic breast cancer

Data retrieved from: https://www.accessdata.fda.gov/scripts/cder/daf/ Retrieval date 04/21/2020 and https://www.cancer.gov/about-cancer/treatment/drugs Retrieval date 04/21/2020. HNSCC—head and neck squamous cell carcinoma, NSCLC—non-small cell lung cancer, SCLC—small cell lung cancer, NET—neuroendocrine tumors, OPFTC—ovarian, primary peritoneal, or fallopian tube cancer, GEJ—gastroesophageal junction, CLL—chronic lymphocytic leukemia, PLL—prolymphocytic leukemia, NHL—non Hodgkin’s lymphoma, AML—acute myelogenous leukemia, MDS—myelodysplastic syndrome, CMML—chronic myelomonocytic leukemia, MPN—myeloproliferative neoplasm, CLL—chronic lymphocytic leukemia, ALL—acute lymphoblastic leukemia.

**Table 2:** Current ongoing trials of unapproved novel agents mainly targeting DNA replication stress.

Mechanism.	Drug	Phase	Details (Including NCT Number)
ATR inhibitor	M-6620/Berzosertib/VE-822/VX-970	I	With cisplatin, and radiation in HPV negative HNSCC(NCT02567422), with standard treatment in esophageal and other cancer (NCT03641547), monotherapy or with carboplatin and paclitaxel in advanced solid tumors (NCT03309150), advanced solid tumors (NCT02157792), with irinotecan in advanced solid tumors (NCT02595931), with WBRT in brain metastasis due to NSCLC, SCLC, or NET (NCT02589522)
		I/II	Carboplatin and gemcitabine in advanced OPPFTC (NCT02627443)
		II	With irinotecan in advanced TP53 mutant gastric or GEJ cancer (NCT03641313), selected tumors (NCT03718091), gemcitabine in recurrent OPPFTC (NCT02595892), cisplatin and gemcitabine in urothelial cancer (NCT02567409), avelumab and carboplatin in PARPi-resistant ovarian cancer (NCT03704467), carboplatin +/-docetaxel in mCRPC (NCT03517969)
	AZD6738	I	HNSCC (NCT03022409), with paclitaxel in refractory cancers (NCT02630199), alone or with radiation (NCT02223923), with AZD9150 or acalabrutinib in refractory NHL (NCT03527147), with gemcitabine in advanced solid tumors (NCT03669601)
		I/II	With carboplatin or olaparib or MEDI4736 in advanced solid malignancies (NCT02264678), with acalabrutinib in CLL (NCT03328273)
		II	In combination with olaparib in SCLC (NCT03428607, NCT02937818), with olaparib in recurrent ovarian cancer (NCT03462342), with olaparib in metastatic triple negative breast cancer (NCT03330847), with olaparib in tumors with mutations in *HDR* genes (NCT02576444), with durvalumab in NSCLC (NCT03334617), with olaparib in selected tumors (NCT03682289), neoadjuvant chemotherapy resistant TNBC (NCT03740893)
	BAY1895344	I	Advanced solid tumors and lymphomas (NCT03188965)
	VX-803/M4344	I	Single agent or in combination with cisplatin, carboplatin or gemcitabine in advanced solid tumors (NCT02278250)
Chk1 inhibitor	LY2606368 (Prexasertib)	I	With cytarabine and fludarabine in AML and high risk MDS (NCT02649764), advanced cancer (NCT02778126, NCT02514603, NCT01115790), refractory solid tumors in pediatric patients (NCT02808650), with ralimetinib in selected cancers (NCT02860780), with cisplatin/cetuximab and radiation in HNSCC (NCT02555644), with olaparib in advanced solid tumors (NCT03057145), with LY3300054 in advanced solid tumors (NCT03495323), with chemotherapy or targeted agents in advanced cancer (NCT02124148), with mitoxantrone, etoposide, and cytarabine in refractory AML and high risk MDS (NCT03735446)
		II	Extensive stage SCLC (NCT02735980), in BRCA1/2 mutated selected cancers (NCT02203513), in solid tumors with replicative stress or HDR deficiency (NCT02873975), refractory ovarian cancer (NCT03414047)
	CBP501	I	With cisplatin and nivolumab in advanced solid tumors (NCT03113188)
WEE1	Adavosertib/AZD1775/MK-1775	I	Advanced solid tumors (NCT01748825, NCT02610075, NCT02482311, NCT03313557), recurrent GBM (NCT02207010), with radiation and temozolomide in GBM (NCT01849146), with olaparib in refractory solid tumors (NCT02511795), with docetaxel and cisplatin before surgery in NSCLC (NCT02508246), with cisplatin and radiation in HNSCC (NCT03028766), radiation and cisplatin in cervical, vaginal or uterine cancer (NCT03345784), pharmacokinetic studies in solid tumors (NCT03333824), with radiation in pontine gliomas in pediatric patients (NCT01922076), with MEDI4736 in solid tumors (NCT02617277), with irinotecan in RAS or BRAF mutated colorectal cancer (NCT02906059), ovarian cancer (NCT02659241), with MEDI4736 in bladder cancer (NCT02546661)
		I/II	With gemcitabine (+Radiation) in pancreatic adenocarcinoma (NCT02037230), with carboplatin in refractory tumors (NCT02813135), with nab-paclitaxel and gemcitabine in pancreatic cancer (NCT02194829), with irinotecan in refractory solid tumors in younger patients (NCT02095132)
		II	Uterine serous carcinoma (NCT03668340), SCLC (NCT02593019), in solid tumors with CCNE1 amplification (NCT03253679), BRCA mutated tumors (NCT02465060), with carboplatin and paclitaxel in squamous cell lung cancer (NCT02513563), with concurrent radiation and cisplatin in HNSCC (NCT02585973), with gemcitabine in OPFTC (NCT02101775), with cisplatin in breast cancer (NCT03012477), in AML, MDS and myelofibrosis (NCT03718143), with chemotherapy in OPFTC (NCT02272790), with olaparib in metastatic triple negative breast cancer (NCT03330847), SETD2-deficient advanced tumors (NCT03284385), with paclitaxel in advanced TP53 mutated gastric cancer (NCT02448329), prostate cancer (NCT03385655), with or without olaparib in recurrent OPFTC (NCT03579316), with olaparib in advanced solid tumors (NCT02576444), with carboplatin in advanced solid tumors (NCT01827384), with carboplatin in extensive SCLC (NCT02937818)
MELK	OTS167	I	Refractory advanced breast cancer (NCT02926690)
		I/II	Refractory AML, ALL, advanced MDS, MPN, CML (NCT02795520)
NEDD8 activating enzyme inhibitor	Pevonedistat/TAK-924/MLN4924	I	Advanced solid tumors (NCT03330106, NCT03486314), with low dose cytarabine in AML and MDS (NCT03459859), with irinotecan and temozolomide in selected tumors (NCT03323034), with ruxolitinib in myelofibrosis (NCT03386214), with decitabine in high risk AML (NCT03009240), with chemotherapy for refractory ALL (NCT03349281), as single agent or with azacytidine in AML and MDS (NCT02782468)
		I/II	Alone or with chemotherapy in mesothelioma (NCT03319537), with azacytidine in AML (NCT03013998), with cytarabine, and idarubicin in AML (NCT03330821)
		II	With azacytidine in refractory AML (NCT03745352), with azacytidine in high risk MDS, CMML or low blast AML (NCT02610777), with azacytidine as maintenance therapy after allogeneic stem cell transplantation for non-remission AML (NCT03709576), with azacytidine in MDS or MDS/MPN after failure of DNA methyl transferase inhibitors (NCT03238248), with docetaxel in NSCLC (NCT03228186), with ibrutinib in refractory CLL and NHL (NCT03479268)
		III	With azacytidine in high risk MDS, CMML or low blast AML (NCT03268954)

Data retrieved from: https://www.accessdata.fda.gov/scripts/cder/daf/ Retrieval date 04/21/2020 and https://www.cancer.gov/about-cancer/treatment/drugs Retrieval date 04/21/2020. HNSCC—head and neck squamous cell carcinoma, WBRT—whole brain radiation therapy, NSCLC—non-small cell lung cancer, SCLC—small cell lung cancer, NET—neuroendocrine tumors, OPFTC—ovarian, primary peritoneal, or fallopian tube cancer, GEJ—gastroesophageal junction, PARPi-Poly (adenosine diphosphate ribose [ADP]-ribose) polymerase inhibitor, mCRPC—metastatic castration-resistant prostate cancer, CLL—chronic lymphocytic leukemia, PLL—prolymphocytic leukemia, NHL—non Hodgkin’s lymphoma, HDR—homologous DNA repair, TNBC—triple negative breast cancer, AML—acute myelogenous leukemia, MDS—myelodysplastic syndrome, CMML—chronic myelomonocytic leukemia, MPN—myeloproliferative neoplasm, CLL—chronic lymphocytic leukemia, ALL—acute lymphoblastic leukemia.
